# Primary Cardiac Diffuse Large B-Cell Lymphoma Managed With Rituximab, Etoposide, Prednisone, Vincristine, Cyclophosphamide, and Doxorubicin (R-EPOCH): A Case Report and Literature Review

**DOI:** 10.7759/cureus.84169

**Published:** 2025-05-15

**Authors:** Jody W Tai, Elliot N Roufeh, Jarred P Reed

**Affiliations:** 1 Internal Medicine, Olive View University of California Los Angeles Medical Center, Los Angeles, USA; 2 Oncology, Olive View University of California Los Angeles Medical Center, Los Angeles, USA

**Keywords:** cardiac lymphoma, cardiac tumors, diffuse large b-cell lymphoma, r-epoch, ventricular tachycardia (vt)

## Abstract

Diffuse large B-cell lymphoma (DLBCL) is the most common subtype of non-Hodgkin’s lymphoma. However, primary cardiac involvement is rare, often presenting with subtle and atypical symptoms that complicate diagnosis and management. We present the case of a 60-year-old man who presented with intermittent palpitations of unclear origin, later identified as ventricular tachycardia. Imaging showed a right ventricular cardiac mass, and biopsy confirmed a diagnosis of DLBCL. He was treated with the chemotherapy regimen rituximab, etoposide, prednisone, vincristine, cyclophosphamide, and doxorubicin (R-EPOCH), resulting in symptomatic resolution of palpitations and a reduction in the size of the cardiac mass. Early detection is crucial to improving survival in primary cardiac lymphomas and highlights the importance of an interdisciplinary approach in managing this rare cardiac malignancy.

## Introduction

Diffuse large B-cell lymphoma (DLBCL) accounts for up to one-third of all non-Hodgkin’s lymphomas [1]. Its incidence increases with age, shows a slight male predominance, and is associated with risk factors such as immunodeficiency (e.g., human immunodeficiency virus (HIV), organ transplantation), chronic autoimmune disorder (e.g., systemic lupus erythematosus), and a personal history of malignancy [1]. DLBCL commonly presents with lymphadenopathy and B-symptoms (fever, night sweats, and/or weight loss). Extranodal involvement is most commonly observed in the GI tract, bone, skin and soft tissues, or genitourinary tract [2]. Cardiac involvement is exceptionally rare, with a reported incidence of only 0.5% among all extranodal lymphomas, primarily documented in case reports [3-5]. Diagnosis can be challenging in the absence of accessible lymph node targets, and treatment carries a theoretical risk of myocardial rupture due to rapid tumor regression. No standardized guidelines exist for the management of cardiac DLBCL, and literature on survival outcomes remains limited. A retrospective analysis of 305 patients with primary cardiac lymphoma (PCL) reported an overall survival rate of 46.6% and a median survival of 45.4 months (interquartile range, 5.62-120.38) [6], suggesting a generally poor prognosis. To our knowledge, only a limited number of cardiac DLBCL cases have been reported as treated with the chemotherapy regimen rituximab, etoposide, prednisone, vincristine, cyclophosphamide, and doxorubicin (R-EPOCH). We present the case of a 60-year-old man with primary cardiac DLBCL successfully managed with R-EPOCH and early interdisciplinary intervention.

## Case presentation

A 60-year-old man with a history of hypertension presented to the emergency department (ED) with palpitations. He reported that the symptoms began three months prior, characterized by intermittent irregular heartbeats noted on his home blood pressure monitor, without associated symptoms. During his initial ED visit, his heart rate was documented between 170 and 179 beats per minute; however, the episode self-terminated before an electrocardiogram (EKG) could be obtained, and no arrhythmia was captured. The patient was then discharged home. One week later, he returned with recurrent symptoms. Telemetry monitoring during his second visit captured a wide-complex tachycardia consistent with ventricular tachycardia. Transthoracic echocardiography revealed a left ventricular ejection fraction (LVEF) of 45% without prior documentation or history suggestive of heart failure. Left heart catheterization showed no evidence of coronary artery disease. He received intravenous antiarrhythmics during hospitalization but was discharged without oral antiarrhythmics for unclear reasons.

**Figure 1:**
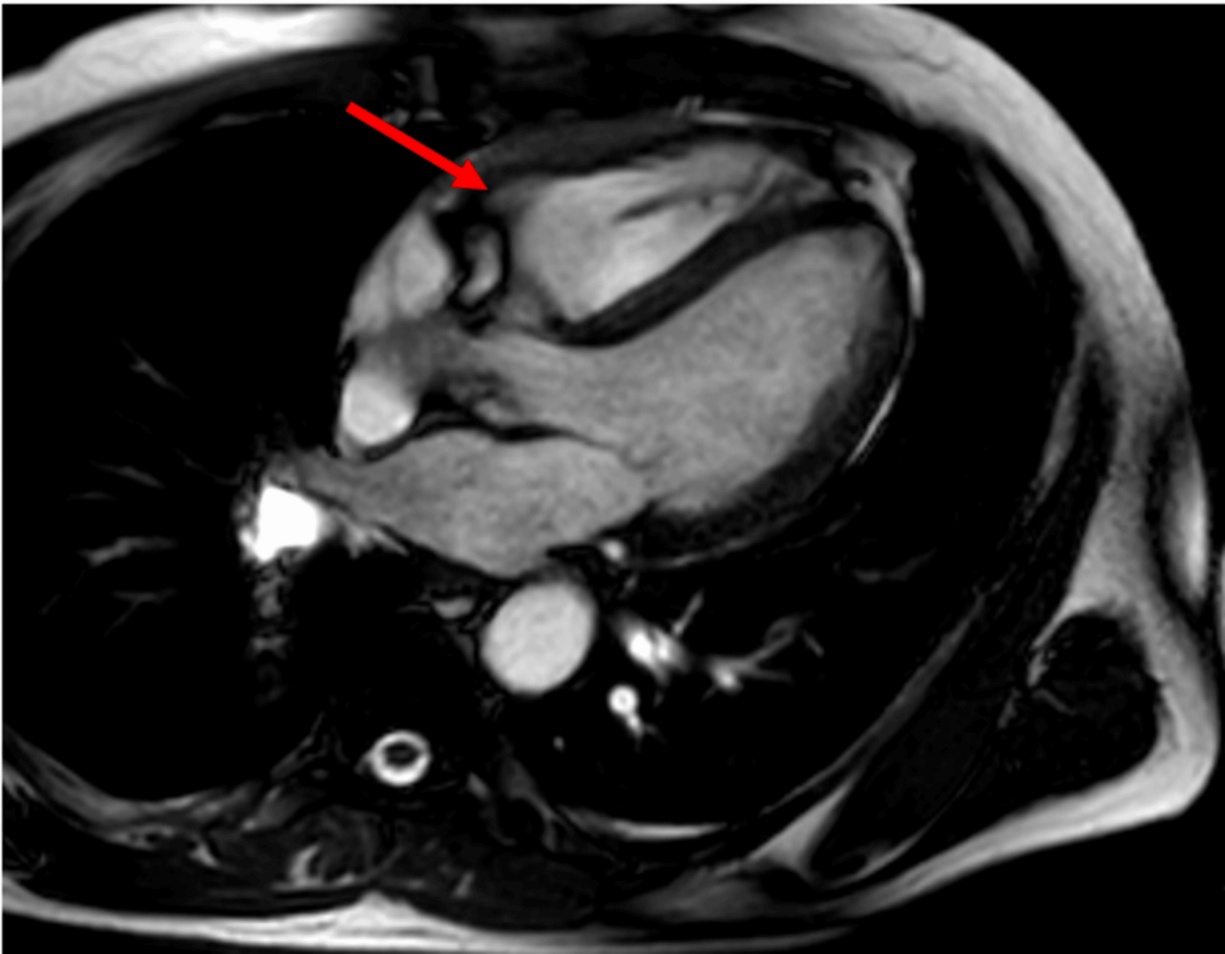
Cardiac MRI showing an ill-defined mass in the right ventricle (red arrow)

Subsequent outpatient cardiac magnetic resonance imaging (MRI) revealed a large, ill-defined, heterogeneously enhancing mass involving the free wall of the right ventricle, extending into the pericardial space with regional hypokinesia (Figure 1).

The patient was advised by his primary care physician to present to our ED for further evaluation, particularly to assess eligibility for advanced therapies not available at the referring facility. This marked his third ED presentation, nearly six weeks after the first ED visit. On arrival, vital signs were notable for intermittent asymptomatic bradycardia with a heart rate in the 30s. Physical exam was unremarkable. EKG showed intermittent premature ventricular contractions, but no sustained ventricular arrhythmias. The patient was admitted for expedited evaluation of the right ventricular mass. Given its heterogeneous appearance on imaging, the differential diagnoses included myxoma, angiosarcoma, or metastasis from other primary malignancies.

**Figure 2:**
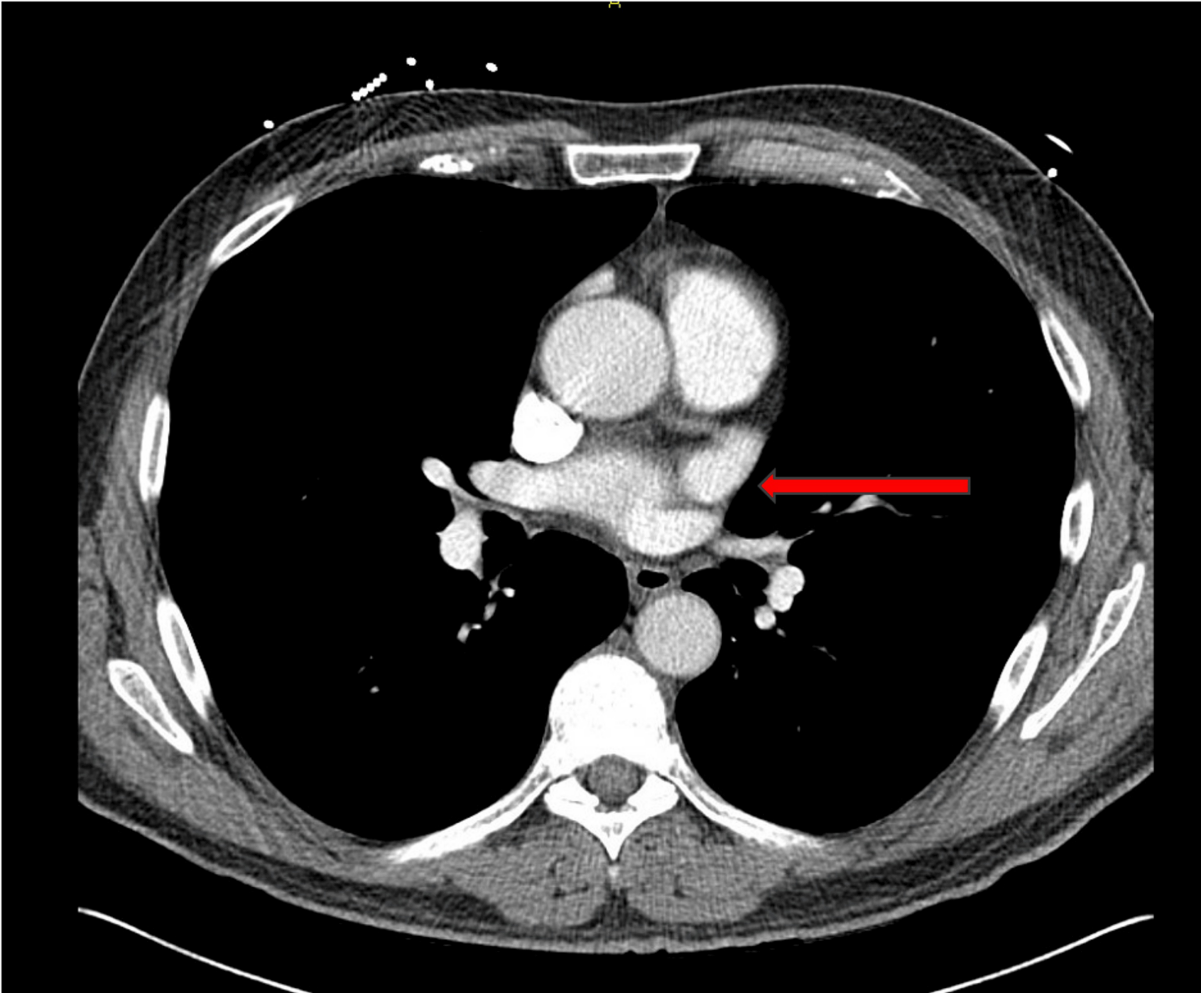
Chest CT showing an enlarged multistation intrathoracic lymph nodes with enlarged right lower paratracheal lymph node, prominent para-aortic lymph node, and a left internal mammary nodal mass concerning for nodal metastasis (red arrow)

A chest computerized tomography scan performed for staging revealed enlarged multistation intrathoracic lymph nodes (Figure 2).

**Figure 3:**
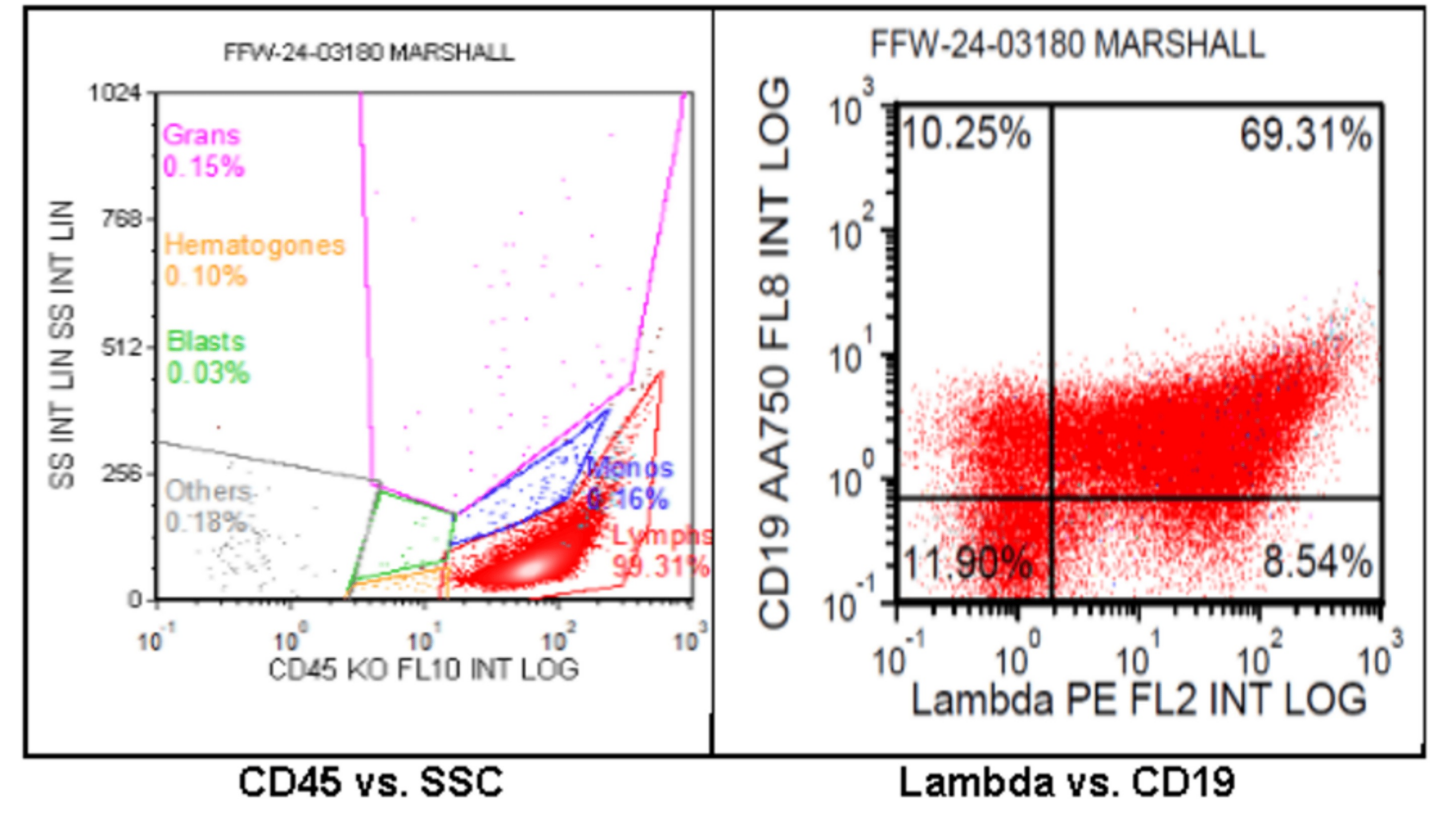
Flow cytometry showing monotypic B-cell population (approximately 80% of the total) positive for CD10 without discrete pan T-cell aberrancy CD: cluster of differentiation.

Abdominal and pelvic CT scans show no evidence of metastatic disease, and brain CT revealed no findings concerning for malignancy. After multidisciplinary consultation with pulmonology, cardiology, oncology, and interventional radiology, a mediastinal lymph node was identified as the most accessible biopsy target. An endobronchial biopsy of lymph node station 2R yielded malignant tissue consistent with germinal center-type DLBCL. The specimen was positive for PAX5, CD19, CD20, CD10, BCL2, BCL6, and MUM1, with lambda light-chain restriction and Ki67 proliferation index of 70%. It was negative for CD3, BCL1, and cMYC. Flow cytometry demonstrated a monotypic B-cell population comprising approximately 80% of total cells, with strong CD10 expression (Figure 3). 

Fluorescence in situ hybridization (FISH) demonstrated IGH-BCL2 fusion [t(14;18)] in 80.5% (161/200) of nuclei examined. No MYC-IGH fusion was detected; however, three copies of the IGH-specific signal were observed in 76% (152/200) of nuclei examined, further supporting the presence of the t(14;18) translocation.

**Figure 4:**
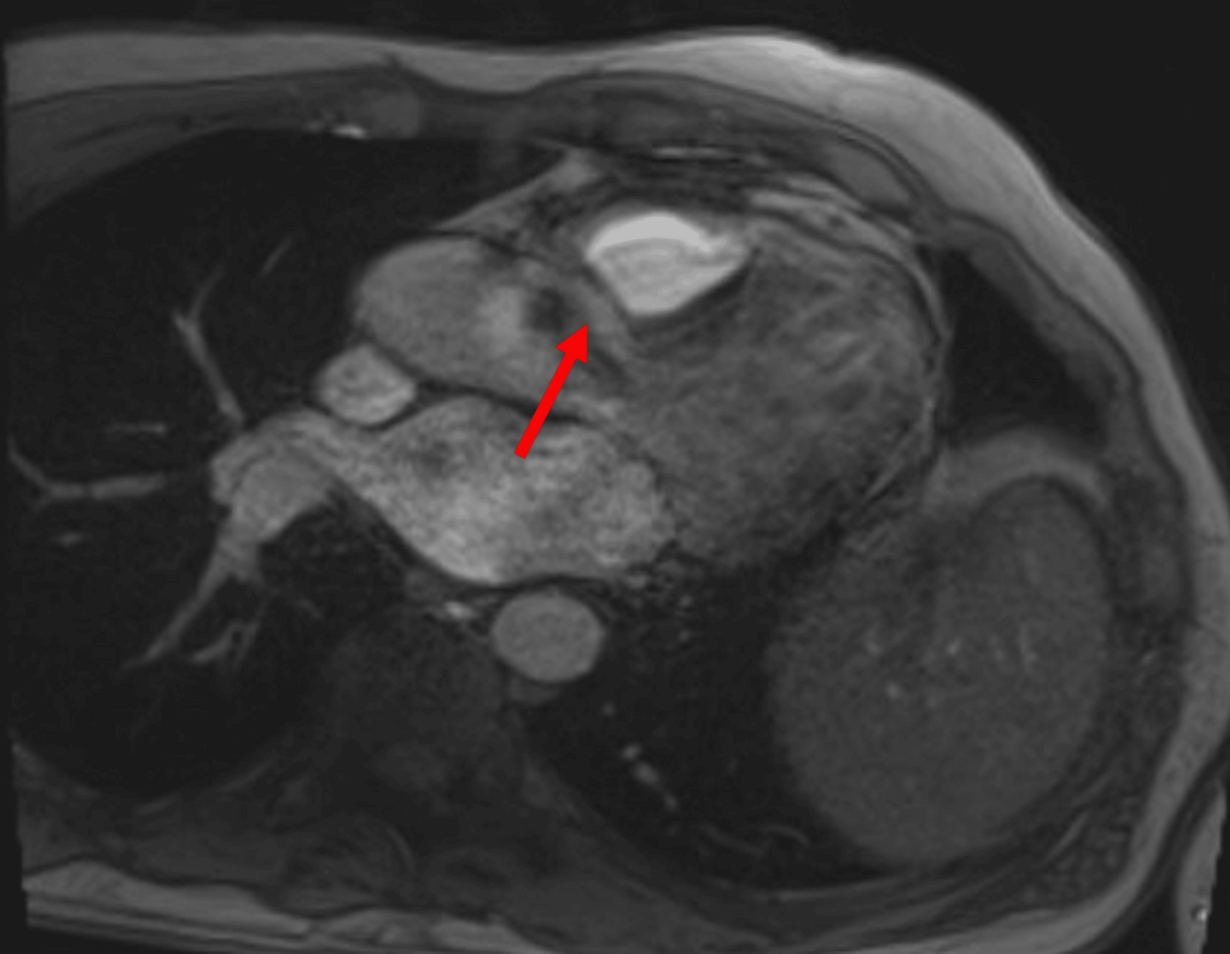
Repeat cardiac MRI showing a mild interval decrease in the right ventricular mass after the first cycle of R-EPOCH R-EPOCH: rituximab, etoposide, prednisone, vincristine, cyclophosphamide, and doxorubicin.

The patient was admitted to the cardiac critical care unit for close monitoring due to the theoretical risk of right ventricular free wall rupture following chemotherapy initiation. Intravenous (IV) amiodarone was initiated for potential ventricular arrhythmias, although only premature ventricular contractions had been observed on telemetry monitoring. Dose-adjusted R-EPOCH (da-R-EPOCH) was initiated, with rituximab given on day 6 instead of the conventional day 1. By hospital day 10, echocardiography revealed a decline in LVEF to 35%, with new-onset LV hypokinesis. Further arrhythmias were suppressed with oral amiodarone, and guideline-directed medical therapy was initiated for heart failure. A follow-up cardiac MRI on day 12 showed a mild interval decrease in the size of the right atrial and right ventricular mass, along with partial recovery of LVEF to 58% (Figure 4). 

**Figure 5:**
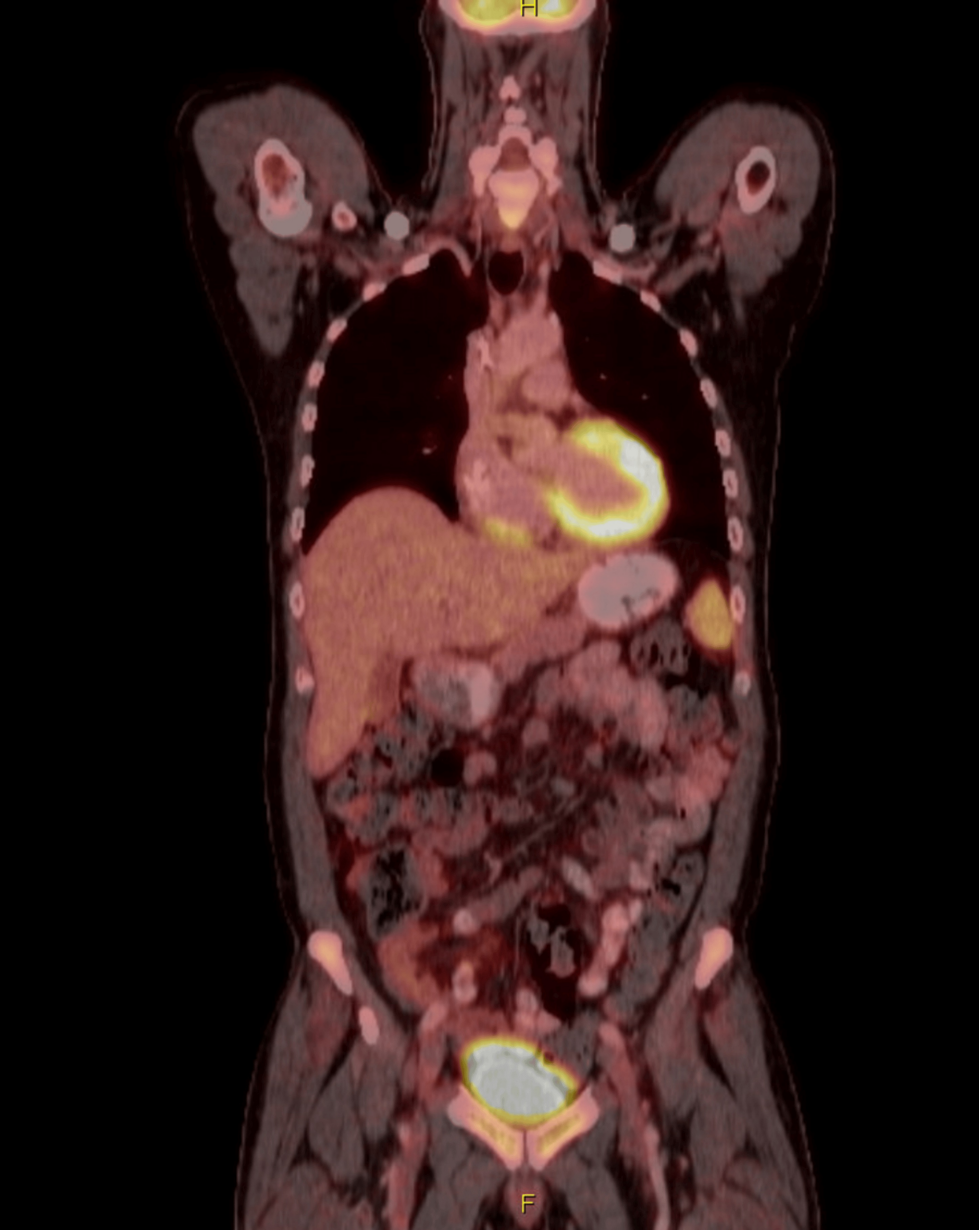
PET-CT after cycle 2 of R-EPOCH chemotherapy Normal physiologic uptake noted in the visualized portions of the myocardium, gastrointestinal tract, and the genitourinary system. No FDG uptake noted in the right atrial infiltrative soft tissue mass. FDG: fluorodeoxyglucose, PET-CT: positron emission tomography-computed tomography, R-EPOCH: rituximab, etoposide, prednisone, vincristine, cyclophosphamide, and doxorubicin.

The patient was discharged and subsequently received a second cycle of da-R-EPOCH. A post-cycle 2 positron emission tomography-computed tomography (PET-CT) scan showed a complete metabolic response (Figure 5). 

As the time of writing, the patient is scheduled to begin cycle 5 of R-EPOCH approximately three months after the initial presentation. Dose adjustment of vincristine was made due to grade I upper extremity neuropathy, and holding doxorubicin is under consideration due to an interval decline in LVEF to 35%-40%. 

## Discussion

Primary cardiac tumors are uncommon, with prevalence rates ranging from 0.3% to 0.7% of all cardiac tumors per autopsy reports [7]. The usual presentation of oncologic involvement of the heart involves metastases to the pericardium due to lymphatic extension or hematogenous spread [8] and are often not large solid masses as seen in this patient. Of these reported solid cardiac tumors, only 25% are malignant, and a further 75% of those are mostly found to be angiosarcomas [8]. For PCLs, DLBCL is the most common subtype, although there have been some case reports of Burkitt and T-cell lymphomas [9,10]. Patients are usually immunocompromised and can present with symptoms such as dyspnea, chest pain, heart failure, or arrhythmias [11]. Even so, making a diagnosis can be challenging due to the nonspecific clinical presentation as well as the accessibility of appropriate biopsy sites. This patient's arrhythmias were not captured on initial presentation, and he was not sent with an outpatient portable device for further cardiac arrhythmia monitoring, so the clinical etiology of his palpitations was unclear until the outpatient cardiac MRI was done. A multidisciplinary discussion with pulmonology, cardiology, and interventional radiology was also required to review the imaging and determine the most accessible and high-yield biopsy site.

Management of primary cardiac DLBCL is complicated by the lack of high-level evidence for the most appropriate systemic therapy due to its rarity. To contextualize overall lymphoma treatment, the R-EPOCH regimen was borne out of a need to intensify and optimize overall survival in poor-risk lymphomas traditionally treated with rituximab, cyclophosphamide, doxorubicin, vincristine, and prednisone (R-CHOP) [12]. For this patient, his Ki67 proliferation index of 70% was an indicator for poor-risk lymphoma [13]. In recent years, strong evidence has suggested that in patients with primary mediastinal DLBCL, R-EPOCH therapy is associated with improved outcomes compared to R-CHOP [14,15]. However, it is unclear if these results can be extrapolated to primary cardiac DLBCL. For the purpose of our literature review, we will exclude primary mediastinal DLBCL and focus only on primary cardiac DLBCL case reports where R-EPOCH has been administered with evidence of success, which is summarized in Table 1 [16-20].

**Table 1:** Compilation of available primary cardiac DLBCL cases treated primarily with R-EPOCH DLBCL: diffuse large B-cell lymphoma, R-EPOCH: rituximab, etoposide, prednisone, vincristine, cyclophosphamide, and doxorubicin.

Author	Year	Study type	Number of patients	Outcome
Rogers et al. [16]	2016	Case report	1	Complete remission after two cycles; chemotherapy discontinued after three cycles due to a decline in performance status
Thiagraj et al. [17]	2018	Case report	1	Complete remission after two cycles; completed six cycles of chemotherapy; remains in complete remission at one-year follow-up
Anand et al. [18]	2019	Case report	1	Complete remission after two cycles; follow-up unknown
Michishita et al. [19]	2024	Case report	1	Complete remission after an unknown number of cycles; completed six cycles of chemotherapy
Rezvani et al. [20]	2024	Case report	1	Complete remission after two cycles; follow-up unknown.

An additional rationale for utilizing R-EPOCH in this setting is the advantage of utilizing infusional therapy, so that the patient can be closely monitored for evidence of arrhythmias via telemetry and serial imaging with echocardiograms for evaluation of cardiac ejection fraction. In this patient's case, the decrease in his LVEF to 35% on day 10, seen on echocardiogram, with subsequent recovery to 58% on day 12, seen on cardiac MRI, was hypothesized to be either due to brief myocardial stunning or a discrepancy in the interpretation between two different imaging modalities. Our literature review also found a mention of a postulated risk of sudden cardiac death due to myocardial rupture from rapid tumor shrinking with chemotherapy administration, although the exact rate of occurrence for this complication is unknown [21]. Regardless, the initial administration of rituximab being given on day 6 instead of day 1 per usual protocol during the patient’s first cycle was done in an attempt to decrease this theoretical risk of myocardial perforation based on extrapolation from a study showing excess rituximab toxicity in HIV-associated non-Hodgkin's lymphoma [22]. Due to the unclear significance of this change, rituximab was given according to usual protocol on day 1 in subsequent cycles. Another aspect of management includes the role of surgery. Unlike other types of primary cardiac tumors, surgery is limited in treating primary cardiac DLBCL tumors due to a lack of positive impact on survival outcomes [23]. However, there are some instances where surgery can be used for debulking or to prevent embolization prior to chemotherapy administration [24,25]. Overall, given the general complexity of managing primary cardiac DLBCL, an early integration of multidisciplinary specialists can be beneficial for diagnosis, treatment, and monitoring.

## Conclusions

Management of primary cardiac lymphoma remains challenging due to its rarity, which contributes to delays in diagnosis and the complexity of high-risk treatment, ultimately resulting in a low five-year survival rate. Our patient benefited from early involvement of multiple specialties, including interventional radiology, pulmonology, and pathology, to identify the safest biopsy target, as well as oncology and cardiology to navigate the intricacies of treatment. His clinical response to da-R-EPOCH highlights the efficacy of this regimen in achieving complete remission. This case reinforces the importance of maintaining a high index of suspicion for cardiac lymphoma in patients presenting with myocardial masses to facilitate early detection and improve outcomes. Looking ahead, the establishment of a standardized national registry could support the development of evidence-based protocols and improve care for patients with this rare malignancy.
